# Correlation between CpG methylation profiles and hormone receptor status in breast cancers

**DOI:** 10.1186/bcr1762

**Published:** 2007-08-31

**Authors:** Weiwei Feng, Lanlan Shen, Sijin Wen, Daniel G Rosen, Jaroslav Jelinek, Xin Hu, Shaoyi Huan, Miao Huang, Jinsong Liu, Aysegul A Sahin, Kelly K Hunt, Robert C Bast, Yu Shen, Jean-Pierre J Issa, Yinhua Yu

**Affiliations:** 1 Department of Experimental Therapeutics, The University of Texas MD Anderson Cancer Center, 1515 Holcombe Blvd, Houston, TX 77030, USA; 2 Department of Leukemia, The University of Texas MD Anderson Cancer Center, 1515 Holcombe Blvd, Houston, TX 77030, USA; 3 Department of Biostatistics, The University of Texas MD Anderson Cancer Center, 1515 Holcombe Blvd, Houston, TX 77030, USA; 4 Department of Pathology, The University of Texas MD Anderson Cancer Center, 1515 Holcombe Blvd, Houston, TX 77030, USA; 5 Department of Surgical Oncology, The University of Texas MD Anderson Cancer Center, 1515 Holcombe Blvd, Houston, TX 77030, USA

## Abstract

**Introduction:**

Aberrant DNA methylation has been found frequently in human breast cancers, associated with the loss of expression of a number of regulatory genes for growth and correlated to clinical outcomes. The present study was undertaken to determine whether methylation of a set of growth-suppressor genes would correlate to the expression of estrogen receptors (ERs) and progesterone receptors (PRs).

**Methods:**

We used a pyrosequencing methylation analysis to study the methylation of 12 known growth-suppressor genes in 90 pairs of malignant/normal breast tissues. We also examined the expression of ERs and PRs in those specimens by immunohistochemistry. Mutations of *p53 *in tumor cells were detected by direct sequencing.

**Results:**

Twelve tumor-suppressor genes: *ARHI*, *RASSF1A*, *HIN-1*, *RARβ2*, *hMLH1*, 14-3-3 σ, *RIZ1*, *p16*, E-cadherin, *RIL*, *CDH13*, and *NKD2 *were selected for this methylation study. Five of them (*RIL*, *HIN-1*, *RASSF1A*, *CDH13*, and *RARβ2*) were frequently methylated in breast cancers (57%, 49%, 58%, 44%, and 17%, respectively) but not the normal breast (0–4%). Two panels of methylation profiles were defined. The methylation of the *HIN-1*/*RASSFIA *panel strongly correlated to the expression of ERs, PRs, and hormone receptors (HRs; which were defined as 'positive' if ERs and/or PRs were positive; *p *< 0.001). Conversely, the methylation of the *RIL*/*CDH13 *panel strongly correlated to negative ER, PR, and HR expression (*p *= 0.001, 0.025, and 0.001, respectively). The subset of triple-negative breast cancers (in other words, those with negative ER, PR, and HER-2/neu status) was positively associated with the methylation of the *RIL*/*CDH13 *panel and negatively associated with the *HIN-1*/*RASSF1A *panel. Mutations of *p53 *were found in nine breast tumors (11%), seven of which lacked methylation in both panels.

**Conclusion:**

We have defined two panels (*HIN-1*/*RASSFIA*, and *RIL*/*CDH13*) of methylation profiles, which correlated, either positively or negatively, to HR status.

## Introduction

Over the past ten years, aberrant DNA methylation has been recognized as one of the most common molecular abnormalities in breast cancer [1,2]. A large body of evidence implicates potential hypermethylation of CpG islands in the loss of expression of a variety of crucial genes. Tumor-suppressor genes with aberrant methylation in breast cancers include *ARHI *[3,4], *RASSF1A *[5], *HIN-1 *[6], the retinoic acid receptor II gene (*RARβ2*) [7], *hMLH1 *[8], 14-3-3 σ [9], *RIZ1 *[10], *p16 *[11], the E-cadherin gene [12], *PTEN *[13], and *BRCA1 *[14]. Methylation in breast cancer has been related to clinical and pathologic characteristics evident at presentation and clinical outcomes. A higher prevalence of *HIN-1 *and *RAR β2 *methylation was found in the lymph nodes, bone, brain, and lung metastases than the primary tumor [15]. Widschwendter and colleagues [16] reported that the methylation of certain genes was associated with hormone receptor (HR) status, in addition to the response to treatment with tamoxifen. A high prevalence of *PGR*, *HSD17B4*, and *CDH13 *methylation has been associated with HER-2/neu-positive breast cancer [17].

Methylation-specific PCR (MSP) is a sensitive assay used to detect methylation and analyze the methylation status of genes of interest. However, problems inherent to this assay (such as those caused by the use of different primers for the methylated or unmethylated alleles, the gel-based data analysis system used, and difficulties in quantitation) have caused frequent false-positive results in tissue-sample analyses. A new technique, pyrosequencing, has been adapted for use in highly sensitive and quantitative methylation analyses [18,19]. Pyrosequencing methylation analysis is a modification of the combined bisulfite restriction analysis (COBRA) that compares favorably with COBRA in sensitivity, specificity, and robustness [18]. Tost and colleagues also confirmed that the pyrosequencing technique is quantitative, amenable to the analysis of bisulfite-treated DNA derived from paraffin-embedded tissue samples, highly reproducible, and accurate [19]. Bisulfite pyrosequencing has been used in clinical trials of hypomethylating drug treatment and provides accurate and reliable results [20].

To investigate methylation profiles in breast cancer cells, we used bisulfite pyrosequencing to screen 12 known tumor-suppressor genes in 90 pairs of breast cancers and normal tissues. Although all 12 genes had been reported to exhibit hypermethylation in a fraction of breast cancer cases, our assays provided a comprehensive survey of their methylation status and confirmed that five genes could be useful in defining a methylation profile in breast cancer cells. Our findings also suggest that two panels of methylation profiles correlated, either positively or negatively, to HR status.

## Materials and methods

### Cell lines

Human breast cancer cell lines SKBr3, MDA-MB-435, MDA-MB-468, BT-20, MDA-MB-231, and MCF-7 were maintained in RPMI 1640 medium supplemented with 10% fetal bovine serum. Normal breast epithelial cells, HMEC 231 and HMEC234, were cultured in a 1:1 solution of MCDB 105 and medium 199 with 15% fetal bovine serum and 10 ng/ml epithelial growth factor (Sigma, St Louis, MO, USA), as described elsewhere [21].

### Tissue samples

We used 90 samples, consisting of paired tissues and associated clinicopathologic data from the Breast Tumor Bank at The University of Texas MD Anderson Cancer Center (Houston, TX, USA). The samples of breast tumors and corresponding adjacent normal-appearing tissues (from tissues located at least 3 cm away from the site at which the tumor was sampled) came from 80 patients who had undergone surgery at The University of Texas MD Anderson Cancer Center in 2004 or 2005 and 10 patients who had been diagnosed with breast cancer between 1995 and 2003. All tissue samples had been fresh-frozen and stored at -80°C. No patient was recruited specifically for this study. All patients gave written informed consent permitting the use of their breast tissue for research at the time specimens were collected. This study was approved by The University of Texas MD Anderson Cancer Center's Institutional Review Board.

### Histopathologic analysis

All breast specimens were reviewed by experienced pathologists. Slides were subject to immunoperoxidase staining for estrogen receptors (ERs; clone 6F11, Novocastra Laboratories Ltd, Benton Lane, UK) and progesterone receptors (PRs; clone1A6, Novocastra Laboratories Ltd), according to the manufacturer's recommendations. Cancers were considered receptor-positive if > 10% of malignant cells showed nuclear staining. Cancers were classified as HR-positive if the ER and/or PR status was positive. *HER-2/neu *gene amplification was assessed by interphase fluorescence *in situ *hybridization (FISH) using the PathVysion *HER-2/neu *probe kit (Vysis Inc., Downers Grove, IL, USA), according to the manufacturer's recommendations. Levels of the *HER-2/neu*:*CEP17 *signal ratio were considered normal if FISH detected ≤ 2.0 copies per cell.

### Microdissection, DNA extraction, and sodium bisulfite treatment

To avoid contamination with normal tissues in the methylation analysis, we isolated breast cancer cells and paired normal breast epithelial cells from tissues by manual microdissection, following the National Cancer Institutes (Bethesda, MA, USA) protocols [22]. In brief, 5–10 μm sections were cut from each archival fresh-frozen tissue block. For each pair of tissues, the presence of tumor cells in malignant tissues and absence of cancer cells in normal tissues were confirmed by histopathologic examination. Frozen tissue sections were fixed with ethanol, stained with H & E, and microdissected using a needle. Each tumor sample contained > 70% tumor cells after microdissection. Genomic DNA was extracted from patient samples, breast cancer cell lines, and normal breast epithelial cells using the Dneasy tissue kit (Qiagen, Valencia, CA, USA). Bisulfite treatment of 1–2 μg of genomic DNA was performed, as previously described [3].

### Genes studied

Twelve tumor-suppressor genes were selected for this study. *ARHI *(CpG I and II), *RASSF1A*, *HIN-1 *(*SCGB3A1*), *RARβ2*, *hMLH1*, the 14-3-3 σ gene, *RIZ1*, *p16 *(*CDKN2A*), and the E-cadherin gene (*CDH1*) were selected on the basis of previous reports of elevated methylation rates in breast cancers [3-12]. *RIL *(*PDLIM4*) [23], *CDH13*, and *NKD2 *were identified as hypermethylated genes using genome-wide methylated CpG island amplification (MCA) from multiple tumors (24). Global methylation was estimated by testing methylation levels of *LINE1 *repetitive elements [25]. We also assessed the methylation of *ERα *and *PGRB *genes.

### Pyrosequencing methylation analysis

Bisulfite pyrosequencing was used to detect methylation of all 15 genes. Pyrosequencing primers were designed using Assay Design software 1.0 (Biotage, Westborough, MA, USA). For each gene, we selected the CpG island region flanking the transcription start site at the 5'UTR. Two to six CpG sites were studied for each particular CpG island. The primers for pyrosequencing and PCR conditions are listed in Additional file 1. Bisulfite-treated DNA (1 μl) was amplified in 50 μl of reaction mixture, containing primers and 0.2 U of Taq polymerase (New England Biolabs, Ipswich, MA, USA). For the amplification of *HIN-1*, *RARβ2*, *hMLH1*, the 14-3-3 σ gene, *RIZ1*, the E-cadherin gene, *NKD2*, and *PGRB*, we used a universal primer approach [18]. The PCR product was purified and methylation was quantitated using the PSQ HS 96A pyrosequencing system and Pyro gold reagents (Biotage). Methylation data are presented as the percentage of average methylation in all observed CpG sites. To set the controls for pyrosequencing, we used cancer cell lines and normal cells that were consistently positive or negative with stable levels of methylation. In this study, each PCR assay included a positive control (the MDA-MB-231 breast cancer cell line, which is highly methylated in most genes) and a negative control (normal breast epithelial cells, HMEC231, which are unmethylated in all genes, except *LINE1*, *ARHI*, and the 14-3-3 σ gene). RKO, a colon cancer cell line that is highly methylated in *hMLH1 *and *p16*, was also used as a positive control.

### *p53 *mutation detection

Genomic DNA was extracted from microdissected tumor tissue using the QIAGEN DNA purification kit (Qiagen). PCR was performed using primers that amplify *p53 *exons 5–6 and 7–9. The PCR products were then purified by the gel purification system (Qiagen) or Exonuclease I and shrimp alkaline phosphatase (USB, Cleveland, OH, USA). Mutations were determined by direct sequencing.

### Statistical methods and analysis

In the studied cohort, adjacent normal breast tissue was taken from each of 90 patients during surgery. Taking advantage of paired normal/tumor samples in this study, we chose the value of normal samples as the reference. If using the sample mean plus two times the standard deviation of the pooled normal samples (and a minimum of 10% methylation) as a cut-off point, there is > 97% probability that the methylation level for a normal tissue will be lower than the cut-off point. It is reasonable to assume that a value larger than the cut-off point is likely to be abnormal (or positive). Panels of genes, that is to say, *HIN-1*/*RASSF1A *and *RIL*/*CDH13 *were considered positive if both markers in each panel were positive. Descriptive analyses were performed first for exploratory purposes. Pair-wise scatter plots are presented to show the correlations among the genes methylated. Heat maps were plotted to show levels of gene expression or methylation using hierarchical clustering to visually represent the association of different genes or samples by histopathologic tumor characteristics (ERs, PRs, and HER-2/neu). Chi-square or Fisher's exact test were used to assess the dependence between two categorical variables. Pearson's correlation coefficient was used to assess the relationship between two continuous variables. The Wilcoxon rank-sum test was used to compare either continuous or categorical variables between two groups. Analysis of variance (ANOVA) was applied to compare the values of gene methylation with tumor characteristics. Multivariate logistic regression was used to assess the ability of various levels of gene methylation to predict the ER, PR, HR, or HER-2/neu status. All reported *p *values are two-sided and considered statistically significant if *p *< 0.05. Analyses were performed using S-PLUS 2000 software (Insightful Corp., Seattle, WA, USA).

## Results

### Patients' clinical data

Patient characteristics are presented in Table 1. Normal paired tissues were unavailable in two cases. Information regarding the grade, HER-2/neu status, and ER and PR status were unavailable for one, ten, and three patients, respectively.

**Table 1:** Patient characteristics

**Clinicopathologic factors**	**Number of sample**
**Age**	90
Median, range	57 years, 29–81 years
**Tumor grade (BNG)**	89
1	3
2	47
3	39
**Tumor size**	90
T1	19
T2	36
T3	18
T4	17
**Stage**	90
I	15
II	37
III	38
**Lymph-node metastasis**	90
pN0	39
pN1	28
pN2	9
pN3	14
**Lymphatic invasion**	78
Positive	33
Negative	45
**Vascular invasion**	78
Positive	32
Negative	46
**ER status**	87
Positive	52
Negative	35
**PR status**	87
Positive	40
Negative	47
**Hormone receptor status**	87
Positive (ER- and/or PR-positive)	55
Negative (both ER- and PR-negative)	32
**HER-2/neu status**	80
Positive	7
Negative	73
**Histology**	90
Ductal	62
Lobular	8
Mixed ductal and lobular	9
Other	11

BNG, Black's nuclear grade; ER, estrogen receptor; PR, progesterone receptor.

### Accuracy, reproducibility, and quantitation of results with bisulfite pyrosequencing

**Figure 1:**
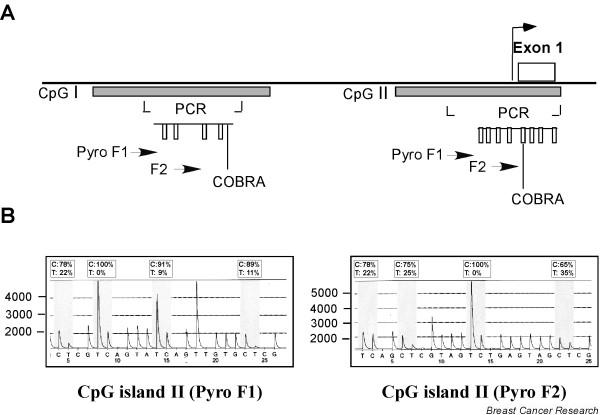
Pyrosequencing analysis for *ARHI *methylation. **(a) **Design of pyrosequencing primers for *ARHI *CpG islands I and II. The vertical bars indicate CpG sites in PCR products. COBRA indicated the restriction enzyme cutting site. **(b) **Pyrosequencing results for *ARHI *CpG island II methylation in MB-MDA-231 cells using two detection primers (Pyro F1 and F2). Methylation levels ranged from 65% to 100%, with a mean of 84%. Gray highlighting marks the CpG sites tested and the percentage reading of C and T. COBRA, combined bisulfite restriction analysis.

To confirm the reliability of bisulfite pyrosequencing for the quantitation of methylation levels, we measured the methylation of *ARHI *CpG islands I and II using pyrosequencing and COBRA and compared the resulting data. COBRA detected only one CpG site, whereas pyrosequencing detected four to eight CpG sites in one CpG island (Figure 1). From six breast cancer cell lines and two cultures of normal breast epithelial cells, methylation data were matched in CpG island II but not completely matched in CpG island I. COBRA showed two breast cancer cell lines were partially methylated in CpG island I (50% and 54% in one CpG site), but they were shown to be hypermethylated in CpG island I by pyrosequencing (the mean of four CpG sites per island was 92% and 91%, respectively). On the basis of these results, pyrosequencing was more sensitive for methylation detection than COBRA.

To confirm the reproducibility of pyrosequencing, the methylation of *HIN-1 *and *RIL *was measured repeatedly in 22 samples using bisulfite treatment and pyrosequencing assays. The correlation between repeated and initial values was high (for *HIN-1*, Pearson's correlation coefficient *R *= 0.99 and *p *< 0.0001; for *RIL*, *R *= 0.99 and *p *< 0.0001). In addition, the variation between individual assays was < 6%: the mean differences and 95% confidence intervals (CIs) for *HIN-1 *and *RIL *were 1.9% with 95% CIs of -0.1% to 3.9% and 3.0% with 95% CIs of 0.1% to 6.0%, respectively.

### Frequent methylation of *RIL*, *HIN-1*, *RASSF1A*, *CDH13*, and *RARβ2*

**Figure 2:**
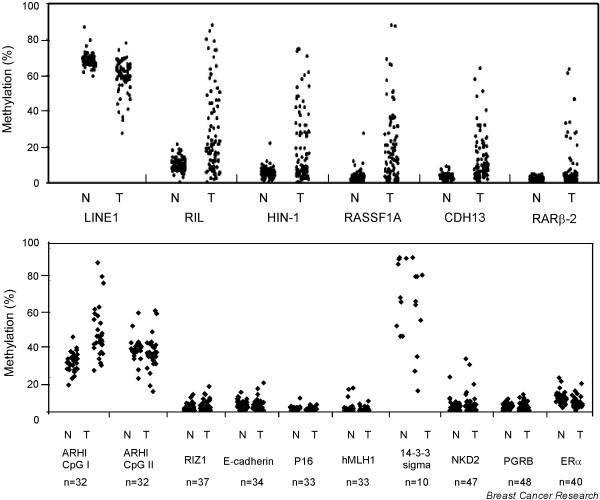
Comparison of promoter methylation levels of 15 genes in paired normal and malignant breast tissue samples.

We evaluated the methylation status of 12 putative tumor-suppressor genes (*ARHI*, *RASSF1A*, *HIN-1*, *RARβ2*, *hMLH1*, the 14-3-3 σ gene, *RIZ1*, *p16*, the E-cadherin gene, *RIL*, *CDH13*, and *NKD2*), *LINE1*, *ER*, and *PGRB *in both culture cell lines and primary cancer tissues. The results from the analysis of six breast cancer cell lines and two normal breast epithelial cell samples (Additional file 2) indicated that 7 of 12 genes (*RIL*, *HIN-1*, *RASSF1A*, *ARHI*, *CDH13*, *RARβ2*, and *NKD2*) were densely methylated (marked bold) in cancer cell lines but not in normal breast epithelial cell lines. We also tested the methylation profiles of the 12 genes in breast cancer and the adjacent normal breast tissues from the same patients. In our pilot study, we screened 12 tumor-suppressor genes in 32–37 paired tissues. Five tumor-suppressor genes (*RASSF1A*, *RIL*, *HIN-1*, *CDH13*, and *RARβ2*) were frequently methylated in breast cancer tissues (the positive rate ranged from 17% to 58%) but not in normal breast tissues (the positive rate ranged from 0% to 4.5%). Another five genes (*RIZ1*, the E-cadherin gene, *p16*, *hMLH1*, and *NKD2*) were not frequently methylated in either malignant or normal breast tissue (the positive rate ranged from 0% to 9%, all < 10%). The 14-3-3 σ gene is highly methylated in both malignant and normal breast tissues. *ARHI*, as an imprinted gene, is at least partially methylated in all cases (Figure 2 and Table 2). Consequently, seven genes were unsuitable for development of methylation profiles. To correlate the methylation profile to clinical outcomes, we focused on the five highly methylated genes by assaying 53 more paired samples. *PGRB *was not frequently methylated in the 48 cases of breast tumor or normal breast tissue. ER was not highly methylated in the 40 cases of breast tumor or normal breast tissue, but higher methylation levels were observed in normal tissues. *LINE1*, a global methylation marker, was highly methylated in all normal and malignant tissues, but the methylation levels were significant lower in tumors (60.0 ± 8.8) than normal tissues (68.2 ± 3.5; *p *< 0.001).

**Table 2:** Methylation levels of 15 genes from pairs of normal and malignant breast tissues

**Gene**	**Number of cases studied**	Methylation level (mean ± SD) (Positive rate*)		
		
		**Normal**	**Cancer**	*p*value**
*LINE1*	90	68 ± 4	60 ± 9	< 0.001
*RIL*	90	10 ± 4 (4.5)	27 ± 22 (57)	< 0.001
*HIN-1*	90	5 ± 3 (2.3)	20 ± 20 (49)	< 0.001
*RASFF1A*	90	3 ± 3 (3.4)	19 ± 20 (58)	< 0.001
*CDH13*	90	3 ± 2 (0)	14 ± 13 (44)	< 0.001
*RARβ2*	90	2 ± 1 (0)	7 ± 12 (17)	< 0.001
*ARHI *CpG I	32	30 ± 6 (0)	47 ± 16 (49)	< 0.001
*ARHI *CpG II	32	38 ± 7 (2)	36 ± 10 (2)	0.250
*RIZ1*	37	2 ± 2 (0)	3 ± 4 (5)	0.111
E-cadherin	34	4 ± 3 (3)	4 ± 4 (9)	0.163
*p16*	33	2 ± 1 (0)	1 ± 1 (0)	0.044
*hMLH1*	33	2 ± 3 (6)	1 ± 1 (0)	0.864
14-3-3 σ	10	75 ± 21	60 ± 28	0.275
*NKD2*	47	2 ± 4 (2)	4 ± 7 (8.5)	0.308
*PGRB*	48	3 ± 2 (0)	2 ± 3 (0)	0.594
*ER*α	40	7 ± 4 (3)	5 ± 3 (5)	< 0.001

*Positive rate using the sample mean plus two times the SD of the pooled normal samples (and minimum 10% methylation) as a cut-off point.

**p value computed using the Wilcoxon signed rank test for paired data. SD, standard deviation.

### Correlation of methylation between different genes and to age

On the basis of data from continuous marker methylation analyses in normal tissues, the methylation levels of *RASSF1A *and *HIN-1 *exhibited a strong correlation to each other (*R *= 0.66, *p *< 0.001). Some moderate correlations were found between *RARβ2 *and *CDH13 *(*R *= 0.40, *p *< 0.001) and between *RIL *and *CDH13 *(*R *= 0.30, *p *= 0.004). Similarly, in tumor tissues, a moderate correlation existed between *RASSF1A *and *HIN-1 *(*R *= 0.51, *p *< 0.001) and a weak correlation existed between *RIL *and *CDH13 *(*R *= 0.19, *p *= 0.068). In addition, the global methylation marker *LINE1 *was negatively correlated to *RIL *(*R *= -0.25, *p *= 0.019) and *RASSF1A *(*R *= -0.26, *p *= 0.014) in breast cancer samples.

In normal tissues, *RIL *methylation levels increased with age (*R *= 0.336, *p *= 0.001), whereas other markers had little correlation to age. In the cancer tissues, *HIN-1 *levels exhibited a weak correlation to age (*R *= 0.229, *p *= 0.03), whereas other markers had little correlation to age.

### Correlation between methylation in malignant and adjacent normal tissues

To study whether gene methylation in cancer would affect adjacent normal-appearing mammary gland tissues, the pair-wise correlation between paired tumor and adjacent normal breast tissues was estimated for each marker. Methylation of *RIL*, *HIN-1*, *RASSF1A*, and *CDH13 *exhibited a positive correlation between breast cancer and normal tissues. The Pearson correlation values ranged from 0.25 to 0.38 (*p *values ranged from 0.02 to 0.0002).

### Relationship between methylation of individual genes and clinical characteristics

Using the ANOVA model, no statistically significant associations were found between clinical stage, tumor size, or node status. Tumors that were poorly differentiated (grade 3) according to Black's nuclear grade (BNG) had lower methylation levels of *HIN-1 *than well- and moderately differentiated tumors (grade 1 + 2; *p *= 0.01). Similarly, *RASSF1A *methylation levels were lower in poorly differentiated cancers than well- and moderately differentiated tumors (*p *= 0.053). In addition, *CDH13 *and *RIL *methylation levels cancers were marginally higher in poorly differentiated than well- and moderately differentiated cancers (*p *= 0.059 and 0.096, respectively).

### Relationship between methylation of individual genes and ER, PR, HR, and HER-2/neu status

In univariate analyses, the ER status was positively associated with high *HIN-1 *and *RASSF1A *methylation levels but negatively correlated to high *RIL *methylation levels (*p *< 0.001, 0.002, and 0.01, respectively). Specifically, patients with a positive ER status had higher methylation levels of *HIN-1 *(mean of 29% in ER-positive tumors versus 9% in ER-negative tumors), whereas patients with a negative ER status had higher methylation levels of *RIL *(mean of 36% in ER-negative tumors versus 22% in ER-positive tumors). In addition, the PR status was positively associated with high *HIN-1 *methylation levels (*p *< 0.001) and negatively associated with high *CDH13 *methylation levels (*p *= 0.03). By defining the HR status as positive if the status of ERs and/or PRs is positive, the associations between the methylation markers and HRs are similar to those between the methylation markers and ERs. In multivariate logistic models, high levels of both *HIN-1 *and *RIL *methylation are independent predictors of the status of ERs and HRs, and high levels of *HIN-1 *and *CDH13 *methylation are independent predictors of PR status (Table 3). Higher methylation levels of *HIN-1 *were more likely to be associated with ER-positive than ER-negative tumors, whereas higher methylation levels of *RIL *were more likely to be associated with ER-negative than ER-positive tumors. Using multivariable logistic models, we have assessed the effect of each gene (*LINE1*, *RIL*, *HIN-1*, *RASFF1A*, *CDH13*, and RARβ2) on HR (adjusted for age), ER (adjusted for age and PR status), or PR (adjusted for age and ER status) status (Additional file 3). The results shown in Additional file 3 are similar to those in Table 3. Among the markers studied, the higher methylation levels of *HIN-1 *were significantly correlated to a lack of *HER-2/neu *amplification (*p *= 0.014).

**Table 3:** Fitted multivariate logistic models for ER, PR, and HR expression

**Single gene model**			**Two-panels model**		
	**Odds ratio (95% CI)**	***p*value**		**Odds ratio (95% CI)**	***p*value**
			
ER			ER		
*HIN-1 *(× 10^-1^)	0.399 (0.25 to 0.63)	0.0001	*HIN-1*/*RASSF1A*	0.06 (0.01 to 0.25)	< 0.0001
*RIL *(× 10^-1^)	1.525 (1.11 to 2.10)	0.0094	*CDH13*/*RIL*	10.14 (2.65 to 38.76)	0.001
PR			PR		
*HIN-1 *(× 10^-1^)	0.551 (0.41 to 0.74)	0.0001	*HIN-1*/*RASSF1A*	0.17 (0.06 to 0.46)	< 0.0001
*CDH13 *(× 10^-1^)	1.674 (1.05 to 2.67)	0.0305	*CDH13*/*RIL*	3.51 (1.17 to 10.51)	0.025
HR			HR		
*HIN-1 *(× 10^-1^)	0.319 (0.18 to 0.57)	0.0001	*HIN-1*/*RASSF1A*	0.05 (0.01 to 0.23)	< 0.0001
*RIL *(× 10^-1^)	1.481 (1.08 to 2.03)	0.0153	*CDH13*/*RIL*	10.18 (2.67 to 38.81)	0.001

CI, confidence interval; ER, estrogen receptor; HR, hormone receptor; PR, progesterone receptor.

### Correlation of two panels of methylation profiles to opposite hormone status

**Figure 3:**
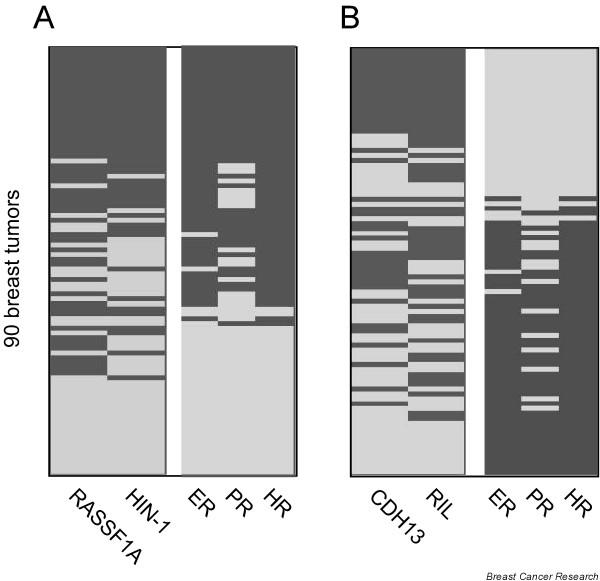
Comparison of two methylation panels with hormone receptor status. **(a) **Dichotomous heat map representing DNA methylation in the *HIN-1*/*RASSF1A *panel (left) and hormone status of each tumor (right). **(b) **Dichotomous heat map representing DNA methylation data in the *RIL*/*CDH13 *panel (left) and hormone receptor status of each tumor (right). Dark gray, positive; light gray, negative.

Because of a correlation between the methylation levels of several of the genes studied, we assembled four potential markers into two panels: *HIN-1*/*RASSFIA *and *RIL*/*CDH13*. A univariate analysis was performed and a multivariate logistic model was fitted to the two panels. The results of the univariate analysis for each panel indicated that the panels had a greater power to predict ER, PR, and HR status (Table 4 and Figure 3) than the methylation of individual genes (Table 3). Methylation of the *HIN-1*/*RASSFIA *panel was strongly correlated to positive ER, PR, and HR expression; 88% of *HIN-1*/*RASSFIA*-positive breast cancers were ER-positive and only 12% of breast cancers were ER-negative (*p *< 0.001). Conversely, methylation of the *RIL*/*CDH13 *panel was strongly correlated to negative ER, PR, and HR expression, because 69% of methylation-positive tumors were ER-negative and only 31% were ER-positive (*p *= 0.001).

**Table 4:** Univariate analysis of clinical variables by *HIN-1*/*RASSF1A *and *RIL*/*CDH13*panels, according to Fisher's exact test

	***HIN-1*/*RASSF1A *panel**			***RIL*/*CDH13 *panel**		
		
	**Number negative (percentage)**	**Number positive (percentage)**	***p *value**	**Number negative (percentage)**	**Number positive (percentage)**	***p *value**
	
**Tumor size**			0.502			0.999
1	11 (20)	8 (24)		14 (22)	5 (19)	
2	20 (36)	16 (47)		25 (39)	11 (42)	
3	12 (21)	6 (18)		13 (20)	5 (19)	
4	13 (23)	4 (12)		12 (19)	5 (19)	
**Tumor grade (BNG)**			0.015			0.162
1 + 2	25 (45)	25 (74)		39 (61)	11 (44)	
3	30 (55)	9 (26)		25 (39)	14 (56)	
**Tumor stage**			0.16			0.745
1	8 (14)	7 (21)		12 (19)	3 (12)	
2	20 (36)	17 (50)		25 (39)	12 (46)	
3	28 (50)	10 (29)		27 (42)	11 (42)	
**Lymph-node metastasis**			0.558			0.78
PN0	24 (43)	15 (44)		26 (41)	13 (50)	
PN1	16 (29)	12 (35)		22 (34)	6 (23)	
PN2	5 (9)	4 (12)		6 (9)	3 (12)	
PN3	11 (20)	3 (9)		10 (16)	4 (15)	
**Lymphatic invasion**			0.244			0.805
1	25 (52)	20 (67)		32 (59)	13 (54)	
2	23 (48)	10 (33)		22 (41)	11 (46)	
**Vascular invasion**			0.346			0.623
1	26 (54)	20 (67)		33 (61)	13 (54)	
2	22 (46)	10 (33)		21 (39)	11 (46)	
**ER status**			< 0.001			0.001
Positive	22 (42)	30 (88)		44 (72)	8 (31)	
Negative	31 (58)	4 (12)		17 (28)	18 (69)	
**PR status**			< 0.001			0.033
Positive	16 (30)	24 (71)		33 (54)	7 (27)	
Negative	37 (70)	10 (29)		28 (46)	19 (73)	
**HR status**			< 0.001			0.001
Positive	24 (45)	31 (91)		46 (75)	9 (35)	
Negative	29 (55)	3 (9)		15 (25)	17 (65)	
**HER-2/neu status**			0.041			0.999
Positive	7 (14)	0 (0)		5 (9)	2 (8)	
Negative	43 (86)	30 (100)		50 (91)	23 (92)	
**Triple-negative**			0.001			< 0.001
No	33 (59)	31 (91)		53 (83)	11 (42)	
Yes	23 (41)	3 (9)		11 (17)	15 (58)	

BNG, Black's nuclear grade; ER, estrogen receptor; HR, hormone receptor; PR, progesterone receptor.

We summarize the fitted multivariate logistic models of the *HIN-1*/*RASSF1A *and *RIL*/*CDH13 *panels by categorical variables (Table 3). Specifically, a patient with a positive value in the *RIL*/*CDH13 *panel is ten times more likely to have an ER-negative tumor than a patient with a negative value. By contrast, a patient with a positive value in the *HIN-1*/*RASSF1A *panel is 17 times more likely to have an ER-positive tumor than a patient with a negative value.

Interestingly, we found that triple-negative breast cancers (in other words, those with negative ER, PR, and HER-2/neu status) were positively correlated to methylation of the *RIL*/*CDH13 *panel and negatively correlated to methylation of the *HIN-1*/*RASSFIA *panel (Table 4). In particular, 58% (15 out of 26) of triple-negative cancers exhibited methylation of the *RIL*/*CDH13 *panel, whereas 17% (11 out of 64) of triple-negative cancers failed to methylate in this panel (*p *< 0.001). Only 9% (3 out of 34) of triple-negative cancers exhibited *HIN-1*/*RASSFIA *panel methylation, whereas 41% (23 out of 56) of triple-negative cancers failed to methylate in this panel (*p *= 0.001). In addition, triple-negative cancers were significantly associated with advanced tumor grade (24% in grade 1 and 2 versus 76% in grade 3; *p *= 0.001).

### Breast tumors with *p53 *mutations lacked methylation

We studied the mutation status of *p53 *in the 84 tumors in which DNA sequences were available for analysis. Overall, *p53 *mutations were found in 9 out of 84 (11%) cases. Seven cases with *p53 *mutations belonged to the group that lacked methylation in both panels; one case was positive in the *HIN-1*/*RASSF1A *panel and one case was positive in the *RIL*/*CDH13 *panel. In the methylation-negative group (both panels were negative), 18% (7 out of 38) of the specimens had *p53 *mutations; in the methylation-positive group (either panel or both panels were positive), only 4% (2 out of 46) of specimens had *p53 *mutation (*p *= 0.072 in Fisher's exact test). Interestingly, five of the cases with *p53 *mutations occurred in triple-negative (ER-, PR-, and HER2-negative) cancers.

## Discussion

Although a large body of evidence has demonstrated that aberrant DNA methylation has an important role in breast carcinogenesis, variation in the data is still a problem. In this study, we used bisulfite pyrosequencing to quantitate the methylation of 12 known tumor-suppressor genes in breast cancers. To avoid contamination with normal tissues, we isolated breast cancer cells by microdissection and compared levels of methylation with those in normal breast epithelial cells from the same patients. Bisulfite pyrosequencing provided sensitive and reproducible measurements. The variation between the different assays was < 6%. Our data confirmed that *RIL*, *HIN-1*, *RASSF1A*, *CDH13*, and *RARβ2 *were frequently methylated in breast cancers but not in normal breast tissues. The other six genes were not highly methylated in breast cancers or methylated in either malignant and normal breast tissues; *ARHI*, as an imprinted gene, has a different methylation status. All the inconsistencies indicate that these seven genes are unsuitable for methylation profile studies.

In the past, methylation data have been correlated to clinical pathologic parameters, to clarify the role of methylation in breast carcinogenesis. Our most interesting finding was a correlation between gene methylation and the HR status. Methylation in breast cancer has already been connected to hormone regulation, but the correlation is not clear yet. Campan and colleagues [26] reviewed the DNA methylation profiles of breast, endometrial, ovarian, and proximal colon cancers but did not find evidence for global hormone-specific DNA methylation alterations. Widschwendter and colleagues [16] reported significant differences in the HR status between clusters of DNA methylation profiles. Their results suggested the existence of an interaction between DNA methylation and HR biology in breast cancer cells. In our own study, we found that the ER status was positively associated with the methylation of *HIN-1 *and *RASSF1A *but negatively correlated to the methylation of *RIL*. In addition, the PR status was positively associated with the methylation of *HIN-1 *and negatively associated with the methylation of *CDH13*. Moreover, if data from the methylation of individual genes were combined into two panels, methylation of the *HIN-1*/*RASSF1A *panel strongly predicted the expression of ERs, PRs, and HRs and methylation of the *RIL*/*CDH13 *panel strongly predicted the negative expression of ERs, PRs, and HRs.

The status of ERs, PRs, and HRs has been recognized as an important prognostic factor in patients with breast cancer, in addition to a predictive marker for the response to treatment with endocrine therapy. The presence of ERs and/or PRs is predictive of the response to treatment with the antiestrogen tamoxifen [27]. ER and PR expression patterns are heavily influenced by changes in the chromatin structure during transcription. Indeed, both the predominant mammalian DNA methyltransferase and histone deacetylases have crucial roles in maintaining transcriptionally repressive chromatin by forming suppressive complexes at replication foci [28]. Our current studies provide evidence that epigenetic changes are tightly connected with HR regulation in breast cancer.

Interestingly, ERs and PRs were not frequently methylated in breast cancers at levels comparable with those observed in the five tumor-suppressor genes. Consequently, alterations in methylation do not seem to have silenced these receptors directly. Increased methylation of certain genes was associated with the expression of ERs and PRs, suggesting that silencing of, at least some, tumor suppressors might affect the transcriptional regulation of HRs, possibly by upregulating HR co-stimulators. Conversely, the downregulation of ERs or PRs might relate to the increased expression of HR co-repressors. These hypotheses will be tested in future studies.

Recently, on the basis of the microarray profiling of invasive breast carcinomas, five distinct subtypes of tumors (luminal A, luminal B, normal breast-like, HER-2/neu overexpressing, and basal) associated with different clinical outcomes have been identified [29,30]. The basal subtype is associated with poor clinical outcomes and the subtype observed in BRCA1-related breast cancers. All basal-like tumors tested in the current study were triple-negative (that is to say, negative for ER, PR, and HER-2/neu expression), poorly differentiated, and high-grade [29,30]. An early study of *HIN-1 *methylation revealed lower frequencies of *HIN-1 *promoter methylation in sporadic breast tumors with a 'BRCA1-like' histopathologic phenotype [31]. In the 90 cases we tested, 76% of the 26 triple-negative tumors were high-grade. Interestingly, we found these tumors to be positively associated with methylation of the *RIL*/*CDH13 *panel but negatively correlated to methylation of the *HIN-1*/*RASSFIA *panel. Our results suggested that methylation reflected by the *RIL*/*CDH13 *panel might have a role in the phenotype of basal-like breast tumors.

Recent advances in molecular biology have revealed numerous genetic alterations involved in breast tumorigenesis; *p53 *mutation is among the most important of those alterations, and studies have reported that *p53 *mutations are strongly associated with poor prognoses in breast cancer [32]. In colorectal cancer, methylation phenotypes define two groups with significantly different genetic lesions (*K-RAS *and *p53 *mutations) [33]. In this study, we have identified 11% of breast tumors that had *p53 *mutations. Equivalent to the observation in colorectal cancers, most cases with *p53 *mutations belong to the group that lack methylation, suggesting that *p53 *mutation and methylation can be two distinct mechanisms that deactivate tumor-suppressor genes in breast cancer. The correlation of *p53 *mutation to hypomethylation is also likely to be owing to the fact that *p53 *mutations and hypomethylation both occur in basal-like triple-negative breast cancers.

The methylation of multiple tumor-suppressor genes has been correlated to poor prognoses in cancers [34]. The use of epigenetic information has shown promise in the identification of patients with gastrointestinal cancers who have poor prognoses [35]. In esophageal carcinoma, the cancers with frequent methylation had significantly poorer survival, and methylation was a better predictor of outcome than the disease stage or patient age [36]. Methylation, as a prognosis factor, has also been described in bladder cancer [37], head and neck cancer [38], ovarian cancer [39], and acute lymphocytic leukemia [40]. In our study, we did not have sufficient survival data to correlate methylation to prognosis (most patients were treated in 2004 and 2005), but we did confirm that the methylation of multiple tumor-suppressor genes was an early event in the subgroups of patients with breast cancer. To understand whether methylation is a prognostic factor in breast cancer, we must conduct studies with longer follow-up times to obtain adequate survival data.

Finally, epigenetic therapy, including the use of demethylating agents (for example, 5-aza-cytidine and 5-aza-2'-deoxycytidine) and histone deacetylase inhibitors (for example, suberoylanilide hydroxamic acid and valproic acid), is currently in clinical trials for myelodysplastic syndrome, leukemia, ovarian cancer, and lung cancer. Previous reports indicate that the 'cross-talk' between inhibitors of DNA methylation and histone deacetylase can result in synergistic activation of silent tumor-suppressor genes in breast cancer. These observations suggest that combination of these inhibitors might be an effective form of epigenetic therapy for breast cancer. It is possible that epigenetic therapy will have a role in the management of breast cancer. The information from this study of methylation profiles will be useful for the study of the biology of breast cancer and refining epigenetic therapy.

## Conclusion

By pyrosequencing methylation analysis, we have examined the methylation profile of 90 normal/breast cancer paired samples. Our data indicated that 5 out of 12 tumor-suppressor genes were frequently methylated in breast cancers but not the normal breast. We have defined two panels (*HIN-1*/*RASSFIA *and *RIL*/*CDH13*) of methylation profiles, which correlated, either positively or negatively, to HR status.

## Abbreviations

BNG = Black's nuclear grade; CI, confidence interval; COBRA = combined bisulfite restriction analysis; ER = estrogen receptor; FISH = fluorescence *in situ *hybridization; H & E = hematoxylin and eosin; HR = hormone receptor; MCA = methylated CpG island amplification; MSP = methylation-specific PCR; PCR = polymerase chain reaction; PR = progesterone receptor; UTR = untranslated region.

## Competing interests

The authors declare that they have no competing interests.

## Authors' contributions

WF, LS, XH, and JJ carried out the methylation assays and helped prepare the manuscript. SH and MH carried out the *p53 *mutation assays. SW and YS performed the statistical analysis. AAS and KKH provided tissues and clinical data. DGR and JL provided pathologic supports for tissue microdisection. YY, JJI, and RCB conceived the study and prepared the manuscript. All authors read and approved the final manuscript.

## Supplementary Material

Additional file 1 Primers and PCR conditions for pyrosequencing assays. Click here for file

Additional file 2 Methylation levels of 12 tumor-suppressor genes, *ERα*, *PGRB*, and *LINE1 *in six breast cancer cell lines and two normal breast epithelial cell cultures. Click here for file

Additional file 3 Multivariable logistic models for assessing effect of each gene (*LINE1*, *RIL*, *HIN-1*, *RASFF1A*, *CDH13*, and *RARβ2*) on HR (adjusted for age), ER (adjusted for age and PR status), or PR (adjusted for age and ER status) status. Click here for file
