# Supplementary material for: Correlation between CpG methylation profiles and hormone receptor status in breast cancers
Source: Breast Cancer Res. 2007 Aug 31;9(4):R57. doi: 10.1186/bcr1762 (PMC2206733; doi:10.1186/bcr1762)
Supplement: Additional file 1 — Primers and PCR conditions for pyrosequencing assays. [file bcr1762-S1.doc]

Supplementary Table 1. Primers and PCR conditions for pyrosequencing assays. All specific sequences are capitalized. The universal tag is in small case.

| Region | Primer sequences and annealing temperature | Product length (bp) | Sequencing primers | Number of CpGs |
| --- | --- | --- | --- | --- |
| LINE1 | Forward:  TTTTGAGTTAGGTGTGGGATATA  Reverse (5’-Biotin):  AAAATCAAAAAATTCCCTTTC  50 °C | 146 | GGGTGGGAGTGAT | 3 |
| RIL | Forward:  TTTGTGAGTTTGGATTGGT  Reverse (5’-Biotin):  CCCCAAATAAACCCTCCAT  58/56/54/52°C | 270 | TTTATTTAGTTTTTAGAGAT | 6 |
| HIN-1 | Forward-Universal:  gggacaccgctgatcgtttaGGGTTTYGYGGAGATAAAGG  Reverse:  CAAAACCACRAAACTTCTTATA-3’  Universal (5’-Biotin)  gggacaccgctgatcgttta  50 °C | 238 | CCTAACCAACTTCCTACTAC | 3 |
| RASSF1A | Forward:  GGGGGAGTTTGAGTTTATTGA  Reverse (5’-Biotin):  CTACCCCTTAACTACCCCTTCC  55 °C | 298 | GGGTYGTATTYGGTTGGAG | 4 |
| CDH13 | Forward:  TTTGGGAAGTTGGTTGGTTG  Reverse (5’-Biotin):  ACAACCCCTCTTCCCTACCT  58°C | 186 | AGGAAAATATGTTTAGTGTA | 6 |
| RARβ2 | Forward:  TTTTGTTAAAGGGGGGATTAG  Reverse-Universal  gggacaccgctgatcgtttaaATTCTCCTTCCAAATAAATACTTAC  Universal (5’-Biotin):  gggacaccgctgatcgttta  55 °C | 161 | TGTTTGAGGATTGGGAT | 5 |
| ARHI  CpG I | Forward:  GTAAGGGAGAAAGAAGTTA  Reverse (5’-Biotin):  TACTATCCTAACAAAACCCT  58 °C | 184 | ATTTGGAAAAGGGATTGG | 2 |
| ARHI  CpG II | Forward:  GTTGGGTTAGTTTTTTATAGTTGGTT  Reverse (5’-Biotin):  AACCAAACAACCTAAAAAACAAATAC  58 °C | 207 | TTGGGGTGTTTAGTTGGTTG | 4 |
| RIZ1 | Forward:  TTTGGGATYGTGGGGAGAT-3’  Reverse-Universal:  gggacaccgctgatcgtttaCCAACRCCTCAAAACACC  Universal (5’-Biotin):  gggacaccgctgatcgttta  55 °C | 204 | GATTGGAGTTAAGATGG | 5 |
| E-Cadherin | Forward:  GGAATTGTAAAGTATTTGTGAGTTT  Reverse-Universal:  gggacaccgctgatcgtttaTCCAAAAACCCATAACTAACC  Universal (5’-Biotin):  gggacaccgctgatcgttta  58 °C | 128 | **GGAAGTTAGTTTAGATTTTA** | 3 |
| P16 | Forward:  GGTTGTTTTYGGTTGGTGTTTT  Reverse (5’-Biotin):  ACCCTATCCCTCAAATCCTCTAAAA  60/58/56/54°C | 175 | **TTTTTGTTTGGAAAGAT** | 2 |
| hMLH1 | Forward:  TTGGTATTTAAGTTGTTTAATTAATAGTTG-3’  Reverse-Universal  gggacaccgctgatcgtttaAAAATACCTTCAACCAATCACCTC  Universal (5’-Biotin)  gggacaccgctgatcgttta  55 °C | 119 | **AGTTATAGTTGAAGGAAGAA** | 2 |
| 14-3-3 sigma | Forward:  TTGGAGGGTGTTGTTTAGTATTGA-3’  Reverse-Universal  gggacaccgctgatcgtttaAATAATCACCCTTCATCTTCAAAT  Universal (5’-Biotin)  gggacaccgctgatcgttta  50 °C | 209 | **GGAGATTGAGTTTTAGGG** | 3 |
| NKD2 | Forward:  TAGGTGTAGGGTTGAGTTTTG-3’  Reverse-Universal  gggacaccgctgatcgtttaAACCATCTTCCCTCACC  Universal (5’-Biotin)  gggacaccgctgatcgttta  60/58/56/54°C | 98 | **TGTAGGGTTGAGTTTTGAA** | 5 |
| PGRB | Forward:  TGTGGGTGGTATTTTTAATGAGA  Reverse-Universal  gggacaccgctgatcgtttaCCCCCTCACTAAAACCCTAAA  Universal (5’-Biotin)  gggacaccgctgatcgttta  58/56/54/52°C | 163 | **GGGATTTGAGATTTT** | 4 |
| ER | Forward:  TGTGTTTTTTTTTTAGGTGG  Reverse (5’-Biotin):  AACCATCCCAAATACTTTAATA  58/56/54/52°C | 125 | GGATACGGTTTGTATTTTG | 3 |
