# Supplementary material for: Correlation between CpG methylation profiles and hormone receptor status in breast cancers
Source: Breast Cancer Res. 2007 Aug 31;9(4):R57. doi: 10.1186/bcr1762 (PMC2206733; doi:10.1186/bcr1762)
Supplement: Additional file 2 — Methylation levels of 12 tumor-suppressor genes, ERα, PGRB, and LINE1 in six breast cancer cell lines and two normal breast epithelial cell cultures. [file bcr1762-S2.doc]

Supplementary Table 2. Methylation levels of 12 tumor suppressor genes, ERα, PGRB, and LINE1 in six breast cancer cell lines and two normal breast epithelial cell cultures. Hypermethylation marked bold.

|  | Tumor suppressor genes | | | | | | | | | | | | |  | Others | | |
| --- | --- | --- | --- | --- | --- | --- | --- | --- | --- | --- | --- | --- | --- | --- | --- | --- | --- |
|  |  |  |  |  |  | ARHI | |  |  |  |  |  |  |  |  |  |  |
| Cell lines | RIL | HIN-1 | RASSF1A | CDH13 | RARβ2 | CpG I | CpG II | RIZ1 | E-cadherin | P16 | hMLH1 | 14-3-3σ | NKD2 |  | LINE1 | PGRB | ERα |
| **Breast cancer** |  |  |  |  |  |  |  |  |  |  |  |  |  |  |  |  |  |
| BT-20 | **94** | **100** | **92** | **80** | **98** | **97** | 19 | 8 | 1 |  | 2 | 0 | **75** |  | 48 | **87** | 2 |
| MCF-7 | **95** | **100** | **100** | **69** | **91** | 50 | 52 | 6 | 2 | deleted | 3 | 0 | 5 |  | 34 | 2 | 0 |
| SKBr3 | **93** | 9 | **100** | 0 | **94** | 40 | 31 | 7 |  | 0 | 2 | **51** | 7 |  | 12 | 0 | 1 |
| MDA-MB-231 | **64** | **79** | **96** | **94** | **63** | **96** | **84** | 16 | **80** | deleted | 3 | 0 | **80** |  | 47 | **61** | **56** |
| MDA-MB-435 | **68** | **61** | **68** | **89** | 0 | **92** | **81** | **85** | **99** | 3 | 3 | **100** | **55** |  | 25 | 31 | **68** |
| MDA-MB-468 | **96** | 35 | **96** | **61** | **92** | **96** | 3 | 6 | 0 |  | 2 | 8 | **63** |  | 49 | 34 | 6 |
| **Normal epithelium** |  |  |  |  |  |  |  |  |  |  |  |  |  |  |  |  |  |
| HMEC231 | 1 | 7 | 0 | 1 | 1 | 46 | 51 | 11 | 4 | 3 | 2 | **97** | 4 |  | **60** | 0 | N/A |
| HMEC234 | 3 | 2 | 2 | 1 | 9 | 39 | 36 | 8 | 6 | 4 | 2 | **96** | 12 |  | **55** | 3 | N/A |
