# Supplementary material for: Correlation between CpG methylation profiles and hormone receptor status in breast cancers
Source: Breast Cancer Res. 2007 Aug 31;9(4):R57. doi: 10.1186/bcr1762 (PMC2206733; doi:10.1186/bcr1762)
Supplement: Additional file 3 — Multivariable logistic models for assessing effect of each gene (LINE1, RIL, HIN-1, RASFF1A, CDH13, and RARβ2) on HR (adjusted for age), ER (adjusted for age and PR status), or PR (adjusted for age and ER status) status. [file bcr1762-S3.doc]

Supplementary Table 3. Multivariable logistic models for assessing effect of each gene (LINE-1, RIL, HIN-1, RASFF1A, CDH13 and RARβ2, respectively) on HR (adjusted for age), ER (adjusted for age and PR) or PR (adjusted for age and ER).

|  | HR |  | ER |  | PR |  |
| --- | --- | --- | --- | --- | --- | --- |
|  | Coefficient (s.d.) | *P** | Coefficient (s.d.) | *P*** | Coefficient (s.d.) | *P*† |
| LINE-1 | -0.02 (0.03) | 0.531 | -0.02 (0.03) | 0.618 | 0.002 (0.03) | 0.964 |
| RIL | -0.03 (0.01) | 0.008 | -0.03 (0.02) | 0.029 | 0.005 (0.01) | 0.714 |
| HIN-1 | 0.11 (0.03) | 0.0002 | 0.06 (0.02) | 0.013 | 0.03 (0.02) | 0.044 |
| RASFF1A | 0.04 (0.02) | 0.023 | 0.04 (0.02) | 0.032 | -0.004 (0.01) | 0.765 |
| CDH13 | -0.04 (0.02) | 0.052 | -0.02 (0.02) | 0.434 | -0.03 (0.02) | 0.161 |
| RARβ2 | 0.003 (0.02) | 0.858 | -0.01 (0.02) | 0.636 | 0.02 (0.02) | 0.489 |

* Adjusted by age: age is not statistically significantly correlated to HR for each of 6 genes, respectively

** Adjusted by age and PR: age is not statistically significantly correlated to ER for each gene, and PR is statistically significantly correlated to ER for all 6 genes

† Adjusted by age and ER: age is not statistically significantly correlated to PR for each of 6 genes, and ER is statistically significantly correlated to PR for all genes
